# The complete chloroplast genome sequence of *Chlorella vulgaris* and phylogenetic analysis

**DOI:** 10.1080/23802359.2020.1787896

**Published:** 2020-07-09

**Authors:** Yangmin Wen, Duanjing Wan

**Affiliations:** a Quanzhou Medical College, Quanzhou, China; b Quanzhou Hospital of Traditional Chinese Medicine, Quanzhou, China

**Keywords:** *Chlorella vulgaris*, chloroplast genome, phylogenetic analysis, genetic information

## Abstract

To understand the process of chloroplast genome evolution, information on repeated sequences, intergenic regions, and pseudogenes in chloroplast DNA is extremely helpful. *Chlorella vulgaris* is a fast-growing fresh-water microalga cultivated on the industrial scale for applications ranging from food to biofuel production. Structure and expression of the chloroplast genome have been studied in a number of plants. Gene content and the sequence of many genes in chloroplast DNA are relatively conserved among land plants and the Euglenophyta Euglena gracilis. The complete chloroplast genome sequence of *C. vulgaris* was characterized from Illumina pair-end sequencing. The chloroplast genome of *C. vulgaris* was 165,412 bp in length and the genome contains no large inverted repeat and has one copy of rRNA gene cluster consisting of 16S, 23S, and 5S rRNA genes. And the genes with two exons containing rrn23 (rRNA), trnl-UAA (tRNA), psba (CDS) and chlL (CDS). The genome contains 114 complete genes, including 78 protein-coding genes (45 protein-coding gene species), 33 tRNA genes (26 tRNA species), and 3 rRNA genes (3 rRNA species). The neighbour-joining phylogenetic analysis showed that *C. vulgaris* and *C. vulgaris*NC001865 clustered together as sisters to other *Salvia* species.

*Chlorella* is a genus of single-cell green algae, belonging to the phylum Chlorophyta. It is abundant in protein, vitamins, unsaturated fatty acids, amino acids, carbohydrate, minerals and fiber. Several species of *Chlorella* have been proposed or have already been used commercially over the past 40 years as a food and feed supplement because of their fast growth and their high resistance to biotic and abiotic stresses. *Chlorella vulgaris* is one of the most cultivated species at the industrial scale because of the high biomass yield and the possibility to grow either in autotrophic or mixotrophic conditions, in the latter case with the addition of reduced carbon source to further improve the biomass yield. *Chlorella vulgaris* has been studied for producing chemicals or heath foods and aquaculture feed, and waste water treatment. *Chlorella vulgaris* is a fast-growing fresh-water microalga cultivated on the industrial scale for applications ranging from food to biofuel production. To understand the process of chloroplast genome evolution, information on repeated sequences, intergenic regions, and pseudogenes in chloroplast DNA is extremely helpful. Therefore, entire nucleotide sequences of green algal chloroplast genomes have been awaited. *Chlorella vulgaris* has high ecological and economic value with high levels of intraspecific genetic diversity. *Chlorella vulgaris* has wide geographic distribution, high intraspecific polymorphism, adaptability to different environments, combined with a relatively small genome size. Consequently, *C. vulgaris* represents an excellent model for understanding how different evolutionary forces have sculpted the variation patterns in the genome during the process of population differentiation and ecological speciation (Neale and Antoine 2011). Structure and expression of the chloroplast genome have been studied in a number of plants. Gene content and the sequence of many genes in chloroplast DNA are relatively conserved among land plants and the Euglenophyta Euglena gracilis. Moreover, we can develop conservation strategies easily when we understand the genetic information of *C. vulgaris*. In the present research, to advance our understanding of its biology and to establish genetics tools for biotechnological manipulation, we constructed the whole chloroplast genome of *C. vulgaris* and understood many genome variation information about the species, which will provide beneficial help for population genetics studies of *C. vulgaris.*

The fresh material of *C. vulgaris* were collected from Quanzhou (118°67′E; 24°54′N). Fresh material was silica-dried and taken to the laboratory until DNA extraction. The voucher specimen (WZ001) was laid in the Herbarium of Quanzhou Medical College and the extracted DNA was stored at −80 °C in the refrigerator of the Key Laboratory of Quanzhou Medical College. We extracted total genomic DNA from 25 mg silica-gel-dried leaf using a modified CTAB method (Doyle 1987). The whole-genome sequencing was then conducted by Biodata Biotechnologies Inc. (Hefei, China) with Illumina Hiseq platform. The Illumina HiSeq 2000 platform (Illumina,San Diego, CA) was used to perform the genome sequence. We used the software MITObim 1.8 (Hahn et al. 2013) and metaSPAdes (Nurk et al. 2017) to assemble chloroplast genomes. We used *C. variabilis* (GenBank: NC015359) as a reference genome. We annotated the chloroplast genome with the software DOGMA (Wyman et al. 2004), and then corrected the results using Geneious 8.0.2 (Campos et al. 2016) and Sequin 15.50 (http://www.ncbi.nlm.nih.gov/Sequin/).

The complete chloroplast genome of *C. vulgaris* (GenBank accession number MT577052) was characterized from Illumina pair-end sequencing. To understand the process of chloroplast genome evolution, information on repeated sequences, intergenic regions, and pseudogenes in chloroplast DNA is extremely helpful. *Chlorella vulgaris* is a fast-growing fresh-water microalga cultivated on the industrial scale for applications ranging from food to biofuel production. Structure and expression of the chloroplast genome have been studied in a number of plants. Gene content and the sequence of many genes in chloroplast DNA are relatively conserved among land plants and the Euglenophyta Euglena gracilis. The chloroplast genome of *C. vulgaris* was 165,412 bp in length and the genome contains no large inverted repeat and has one copy of rRNA gene cluster consisting of 16S, 23S, and 5S rRNA genes. And the genes with two exons containing rrn23 (rRNA), trnl-UAA (tRNA), psba (CDS) and chlL (CDS). The genome contains 114 complete genes, including 78 protein-coding genes (45 protein-coding gene species), 33 tRNA genes (26 tRNA species) and 3 rRNA genes (3 rRNA species).

We used the complete chloroplast genomes sequence of *C. vulgaris* and 8 other related species to construct phylogenetic tree. The 9 chloroplast genome sequences were aligned with MAFFT (Katoh and Standley 2013), and then the neighbour-joining tree was constructed by MEGA 7.0 (Kumar et al. 2016) (Figure 1). The neighbour-joining phylogenetic analysis showed that *C. vulgaris* and *C. vulgaris*NC001865 clustered together as sisters to other *Chlorella* species.

**Figure 1.**
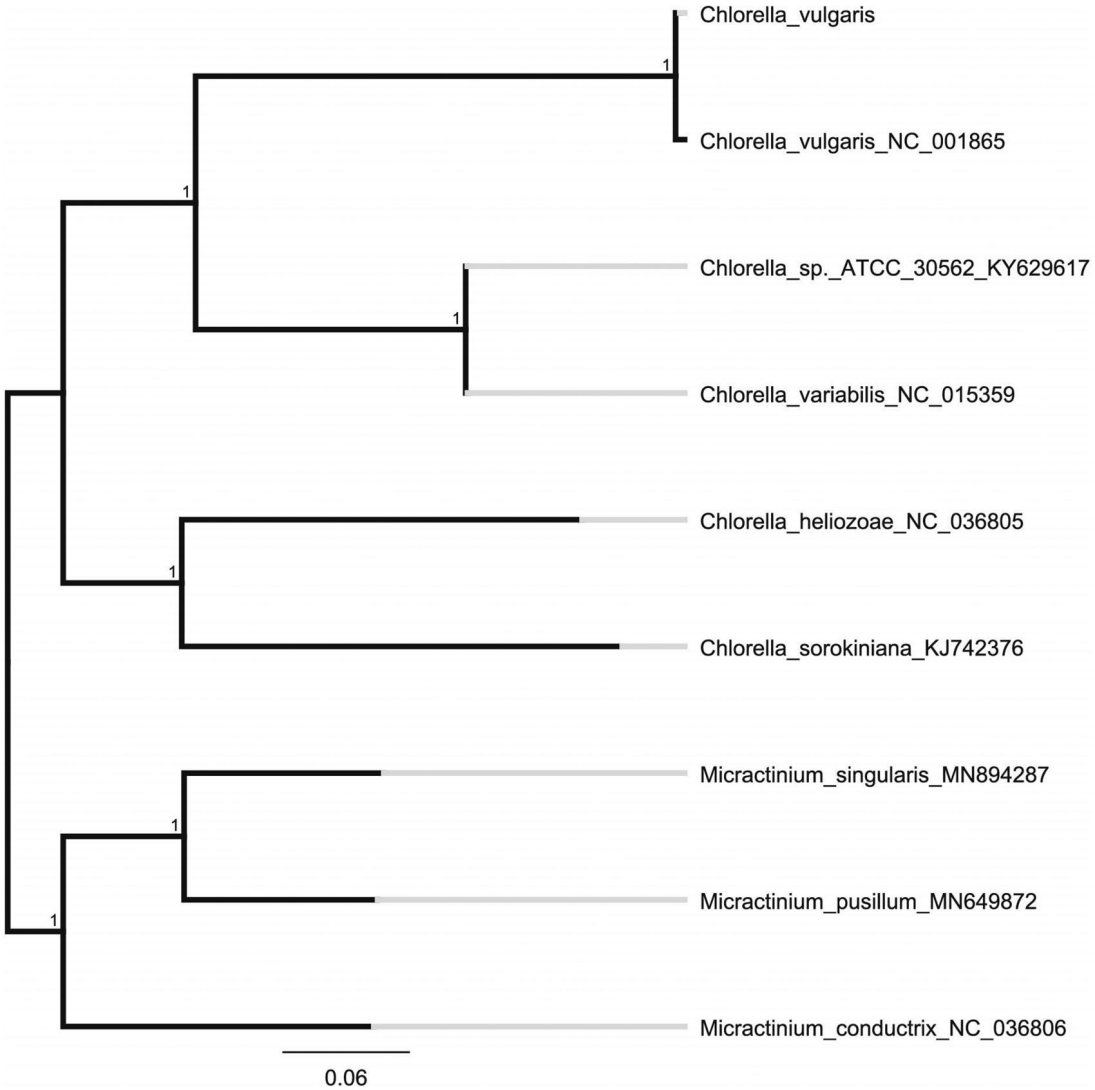
Neighbour-joining (NJ) analysis of *C. vulgaris* and other related species based on the complete chloroplast genome sequence.

## Data Availability

The data that support the findings of this study are openly available in GenBank at https://www.ncbi.nlm.nih.gov, reference number MT577052.
